# Deeper insight into the role of IL-17 in the relationship beween hypertension and intestinal physiology

**DOI:** 10.1186/s12950-022-00311-0

**Published:** 2022-10-04

**Authors:** Ze-Jun Yang, Tian-Tian Wang, Bo-Ya Wang, Han Gao, Cheng-Wei He, Hong-Wei Shang, Xin Lu, Ying Wang, Jing-Dong Xu

**Affiliations:** 1 Clinical Medicine of “5+3”program, School of Basic Medical Science, Capital Medical University, Beijing, China; 2 Department of Cardiology, Beijing An Zhen Hospital, Capital Medical University, Beijing, China; 3 Department of Physiology and Pathophysiology, School of Basic Medical Sciences, Capital Medical University, Beijing, China; 4 Eight Program of Clinical Medicine, Peking University People’s Hospital, Beijing, China; 5 Morphological Experiment Center, School of Basic Medical Sciences, Capital Medical University, Beijing, China; 6 Department of Dermatology, Beijing Tongren Hospital, Capital Medical University, Beijing, China

**Keywords:** Immune factors, IL-17, Gut microbiota, Hypertension, MCP-1, TGF-β, IL-6, IL-10

## Abstract

With the incidence of hypertension increasing worldwide, more and more the mechanisms of hypertension from the perspective of immunity have found. Intestinal microbiota as well as its metabolites relationship with hypertension has attracted great attention from both clinicians and investigators. However, the associations of hypertension with lesions of a large number of immune factors including IL-17, MCP-1, IL-6, TGF-β, IL-10 and others have not been fully characterized. In this review, after introducing the immune factors as the most potent anti/pro-hypertension agents known, we provide detailed descriptions of the IL-17 involved in the pathology of hypertension, pointing out the underlying mechanisms and suggesting the clinical indications.

## Highlight

1. In this review, after summarizing the general roles of intestinal physiology in the pathology of hypertension in detail, we point out the potential use of metabolism of intestinal flora and immune factors in its treatment.

2. We put the interactions of hypertension and a wide-array of cytokine together with metabolites of intestinal flora and intestinal cells, and show the limitations of present researches.

3. We give specific introduction to recent findings of MCP-1, IL-6, TNF-β and IL-10 in hypertension and show underlying effect in the pathogenesis of effects on kidney, cardiovascular and other tissues.

4. As the well-recognized pivotal control of hypertension, interleukins in the mediation of signals transmitted through cardiovascular from digestive tract, whose mechanisms remain largely covered and called for more investigations to address the important issue.

## Introduction

The intestine is an important digestive organ as well as a significant immune organ. The lymphoid tissues of the intestinal tract, such as Peyer patch, could respond to foreign or autogenic antigens and have an important effect on immune surveillance and mucosal immunity [1]. Hypertension is a complex disease caused by multiple physiological changes, and can lead to several systems damage [2–4]. The pathology of hypertension as well as its relationship with the gut has become a new hot-spot research for scientists [5–7]. Especially in recent years, a growing number of researchers have revealed the important roles of numerous immune factors such as Interleukin (IL)-17 during the development of hypertension [8, 9]. Research indicated that neutrophil cytoplasts can promote Th17 inflammation [10]. Neutrophils may induce vasculature damage, infiltrate the kidneys and induce target organs damage [11]. Besides, researchers put forward the “enterotype”, which refers to stratification of human gut microbiota, including *Bacteroides*, *Prevotella* and *Ruminococcus* [12, 13]. *Prevotella* may play an essential role in hypertension, probably by triggering the inflammatory response [14]. The bile acids, a kind of metabolite of intestine, are also shown to decrease blood pressure through G protein-coupled bile acid receptor (TGR5) [15]. A systematic review of observational studies indicated that poor absorption and high excretion of short-chain fat acids (SCFAs) may play an important role in the pathogenesis of hypertension [16]. These evidence associate intestinal physiology with hypertension and thus can become potential therapeutic targets or diagnostic markers. Observational data shows that hypertensive rodents and humans manifest gut dysbiosis, as seen by reduced microbial abundance, richness, and diversity and clear taxonomical distinction [17–19].

It’s true that the study about direct correlation between intestinal microbiota, IL-17 and hypertension is insufficient [20]. However, observations have indicated that the IL-17 could be a contributing factor to hypertension and was affected by the diversity of intestinal flora [20]. Evidence suggests that IL-17 is among the most important factors for immune regulation. In this way, the potential relationship deserve further studying, while the immune factors like IL-17 might be the important milestone point. The purpose of the paper is to review recent research into the potential role of the immune factors in hypertension and the prospective of gut microbiota.

### Role of immune system in regulation of hypertension

Hypertension as a common cardiovascular disease has been widely studied, and it is shown that hypertension is the consequence of multiple factors and can cause multiple organ damage. Immune system is one of the important factors and raise concern.

#### Immune system and hypertension

The past decade has seen the rapid development of the comprehension towards the immune system, a powerful defense against diseases lies in the distinction it makes between the “self” and the “none-self”. However, “The danger model” made by Prof. Polly Matzinger extends the nature of immune system, suggesting that immune system may be more concerned about the things that can do harm to our body [21]. Extensive research has shown that the alarm signals produced by injured cells could activate immune system and initiate chronic inflammation which could then lead to cardiovascular diseases or hypertension [22]. Based upon the generalizability of much published research on this issue, it is generally accepted the concept that the numerous cytokines and metabolites in gut can be regarded as alarm signals contribute to promoting and protecting public health, which could eventually lead to inflammation and hypertension (for detail see box 1).

By employing qualitative model of biomolecular and cells like IFN-γ, IL-10, IL-17 and Th17 cells, scientists have put forward a brain-gut-bone marrow axis to illuminate the regulatory network of hypertension on a comprehensive scale [23]. The importance and originality of this summary are that it highlights the roles of gastrointestinal tract, neuro-inflammation and the growth of immune cells in bone marrow. And existing body of research on hypertension suggests that it has sharp positive association with some autoimmune and inflammatory disease [24]. Notably, the predictive value of some inflammation biomarkers like C-reactive protein (CRP) for the incidence of hypertension or the clinical outcomes has been explored in several studies [25–27]. The recent study also demonstrated the role of hypertension in the delay of viral clearance and airway hyper-inflammation exacerbation in patients with COVID-19 [28], indicating the potential association between hypertension and immune system.

#### Roles of IL-17 in intestinal phyiology/pathology

In 1995, IL-17 receptor A (IL-17RA) was found to be part of a brand new cytokine receptor family [29, 30]. The IL-17 receptor family now consists of 5 members (IL-17RA, RB, RC, RD and RE), all of which, like their ligands, share sequence homology. IL-17RA is ubiquitously expressed on a wide range of tissues and cells [31].

As for the Th17 cells differentiation, after TCR activation and co-simulation, TGF-β and IL-6, which activate transcription factors Smads and STAT3 respectively, induce the expression of Th17 polarized transcription factor retinoid-related orphan receptor (ROR) γt to initialize Th17 differentiation from naive T cells [32–34]. IL-21, which is produced by Th17 cells, further promotes this process in a positive feedback manner [32]. Other regulatory cytokines and transcription factors like IL-1β, IL-2, IL-23 and Pyruvate kinase M2 are also involved in the differentiation of Th17 cells in recent years [35–37]. Nowadays, it has been found that intestinal microorganisms can promote the production of IL-6 by inducing dendritic cells (DC) and Mφ, and IL-6 plays an essential role in regulating the effect of intestinal microorganisms on intestinal inflammation [38, 39]. The supplement of Th17 cells differentiation is listed in Box 2.

**Fig. 1:**
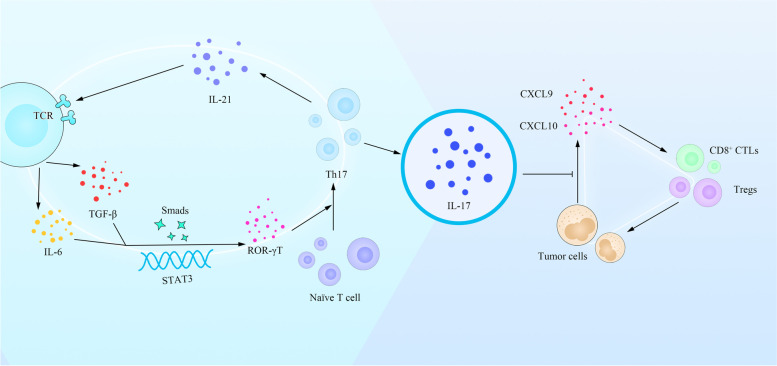
Diagram of the generation of Th17 cells and the association with other factors. While TCR is activated, TGF-β and IL-6 activate transcription factors *Smads* and *STAT3* and induce the expression of ROR, so that naive T cells differentiate to Th17, which secretes IL-21 and in turn promotes the process in a positive feedback manner continually. IL-17 inhibits the production of CXCL9/10 from tumor cells, thus stop CD8^+^ CTLs and Tregs from infiltrating tumor cells

IL-17 signals to colorectal tumor cells and inhibits their production of CXCL9/10 chemokines, thus inhibiting the infiltration of CD8^+^ CTLs and Tregs to colorectal tumor cells [40]. Increased expression of IL-17 family members and the presence of IL-17-producing cells have been reported to relate with Crohn’s disease and ulcerative colitis, while IL-17F and IL-17RA have an increased susceptibility to commensal fungi mainly manifesting as mucocutaneous candidiasis though enhanced expression of IL-17 family members and the IL-17secretion [41]. A Syk-kinase-coupled signaling pathway in DCs was critical for commensal-dependent production of IL-17 by CD4^+^ T cells [42]. Research also showed that Th17 cells controlled segmented filamentous bacteria (SFB) burden, because disruption of IL-17R signaling in the enteric epithelium resulted in SFB dysbiosis [43]. SFB are commensal organisms that grow by anchoring a specialized holdfast structure to the intestinal walls of a variety of animals including monkey, mouse, rat [44, 45]. Importantly, the qPCR and metagenomics analyses of the luminal fluids revealed SFB is a rare member of microbiota in human [46]. In this way, their role in human hypertension related mechanisms may need more researches (Fig. 1).

##### Box 1 immune and hypertension

Numerous immune cytokines can be seen as alarm signals released from body and cause inflammation and hypertension.

A brain-gut-bone marrow axis has been proposed to explain the regulatory network of hypertension.

The cytokines guiding the development of Th17 cell lineage may include IL-6, IL-21, IL-23 and IL-1-β.

##### Box 2 Th17 cells differentiation

Th17 cells, which represent a completely distinct subset of CD4^+^ T cells, produce IL-17A and IL-17F and express the IL-23 receptor and RORγt.

The differentiation of Th17 cells is influenced by cytokines such as TGF-β, IL-1β, IL-12, IL-23 [47–50].

Mφ involved in local inflammation induced the differentiation of Th17 cells in response to Th17-promoting cytokines, and the number of M1Mφ, M2Mφ and Th17 cells increased as the lung inflammation was aggravated. But the relationship between different types of macrophages and the differentiation of Th17 cells in hypertension remains blurry [51, 52].

### Immune factors between immune system and hypertension influenced by gut

A variety of immune organs, cells and cytokines make up the complicated immune system. And numerous studies have demonstrated that immune cytokines have close interactions with each other and build a regulatory network between gut and hypertension. The role of immunity has been examined in experimental hypertension induced by a variety of maneuvers [53]. Here we review roles of several important immune cytokines related with hypertension so as to clarify their vital roles.

#### IL-17 as the vital regulatory immune factor in hypertension

IL-17 is a proinflammatory cytokine found in 1995 through cloning cDNA encoding human IL-17 from CD4^+^ T cells library [54]. The IL-17 family is a subset of cytokines consisting of IL-17A-F, among which IL-17A is the most widely investigated [31, 55]. CD8^+^ cells have also been shown to produce this cytokine, these are called “Tc17” [56]. IL-17^+^ γδ T cells (γδ17 T cells), natural killer cells, and T helper 17 (Th17) cells are the well-known providers of IL-17A in various models of inflammatory diseases [57, 58]. IL-17^+^ γδ T cells can directly interact with pathogens via TLR and dectin-1, leading to the release of IL-17 [59]. Here we focus on the Th17 cell because of its relationship with other immune factors. IL-17A is shown to be one of the major drivers for several inflammatory and autoimmune diseases like multiple sclerosis, psoriasis, asthma, Crohn’s disease and rheumatoid arthritis [31]. Besides, IL-17F and IL-17A are co-expressed on linked genes and co-produced by Th17 cells [60]. IL-17 and IL-17F exist as homodimers but can also be produced as an IL-17AF heterodimer, and they induce signals through an obligate dimeric IL-17RA and IL-17RC heterodimer [20]. Study also indicated IL-17F levels contribute to spontaneously hypertensive rat (SHR) hypertension [61]. A recent study has also found a rising number of Th17 cells in patients suffering from hypertension [62].

IL-17 can promote the activation of T cells and initiate the secretion of inflammatory cytokines like IL-6, IL-8, granulocyte colony stimulating factor (G-CSF), monocyte chemoattractant protein (MCP), CXC chemokines (CXCL)-8 and CXCL-10 from endothelial cells, fibroblasts and macrophages (Mφ) [63, 64]. In the meantime, it synergistically amplifies the effects of inflammatory responses by interacting with IL-1β, IL-22, IFN-γ, and TNFα [65, 66]. The most noteworthy feature of the IL-17 is that it also involved in many diseases, such as inflammatory bowel disease (IBD), rheumatoid arthritis, psoriasis and airway inflammation, and recent studies have shown its proinflammatory effects related to the development of hypertension in terms of intestinal physiology [9, 67].

As is known to all, the renin–angiotensin system (RAS) plays a variety of physiological and pathological roles, and the RAS components existed in intestinal epithelial and immune cells, which may be involved in the regulation of intestinal absorption of fluids and electrolytes [68]. Angiotensin (Ang) II could promote the T cell proliferation and therefore have a regulatory effect on hypertension [69, 70]. In order to confirm the conclusion, salt-sensitive hypertension in Dahl salt-sensitive (SS) rats with a deficit in T and B lymphocytes due to a mutation in the Rag1 gene (SS-Rag1em1Mcwi) was performed to assess, finally demonstrating the importance of renal infiltration of immune cells in the pathogenesis of hypertension and renal damage in Dahl SS rats [71]. Some researchers put forward the hypothesized model that excess dietary salt alters the gut microbiome and activates DCs, thus promoting T cell activation and stimulating the release of IL-17, TNF-α and IFN-γ, leading to hypertension [72]. Researchers found that IL-17^−/−^ mice showed no sustained hypertension compared with wild type mice by 4 weeks of AngII infusion [73]. They also manifested the decrease of superoxide and the aortic T cells in response to AngII. In the meantime, some investigations further elucidated the mechanism through which IL-17 promotes hypertension [74]. It was found that IL-17A could induce the serum/glucocorticoid regulated kinase 1 (SKG1) expression in the kidney and affect the sodium transporter in the distal tubules of the kidney [35]. This could in turn lead to enhanced salt, water reabsorption and elevated blood pressure. Studies also indicate that expression of SGK1 in CD11c^+^ APCs contributes to the pathogenesis of salt-sensitive hypertension [75]. Some studies have reported that IL-17 infusion to C57BL/6 mice significantly enhanced systolic blood pressure and reduced NO-mediated vasodilation [76]. Furthermore, IL-17 is also able to increase RhoA/Rho-kinase-mediated endothelial nitric oxide synthase residue threonine 495 (eNOS Thr495) phosphorylation and further leads to hypertension and decreased NO-mediated vasodilation [76]. Although SFB may regulate the IL-17, whether this effect can pass to NO production remains unclear. In mice, systemic infusion of IL-17A increased blood pressure and induced kidney inflammation, showing increased infiltration of inflammatory cell like T-lymphocytes, neutrophils, monocyte/macrophages and mastocytes [77].

Intestinal microbiota can regulate hypertension by affecting Th17 cells and IL-17. Importantly, a recent study revealed that increased blood pressure induced by dysbiotic microbiota is affected by the T cell activation that requires co-stimulation via B7 ligands [78]. On the other hand, the metabolites of intestinal microorganisms such as SCFAs have been shown to have certain anti-inflammatory effects in intestinal physiological state [79–82]. N-butyric acid salt regulates the function of T cells by inhibiting histone deacetylase (HDAC), which promotes the acetylation of kinase p70 S6 and phosphorylated rS6. This could further activate the mTOR pathway necessary for the proliferation of Th17, Th1 and IL-10^+^ T cells and result in a higher level of IL-17, IFN-γ, and IL-10 [80, 83]. At the same time, the adhesion of SFB, which is common in many vertebrate intestines such as mice, rats, chickens and humans, could also induce the production of serum amyloid A protein (SAA) and reactive oxygen species (ROS) in intestinal epithelial cells, which may further lead to the development of antigen specific Th17 cells [84–87].

**Fig. 2:**
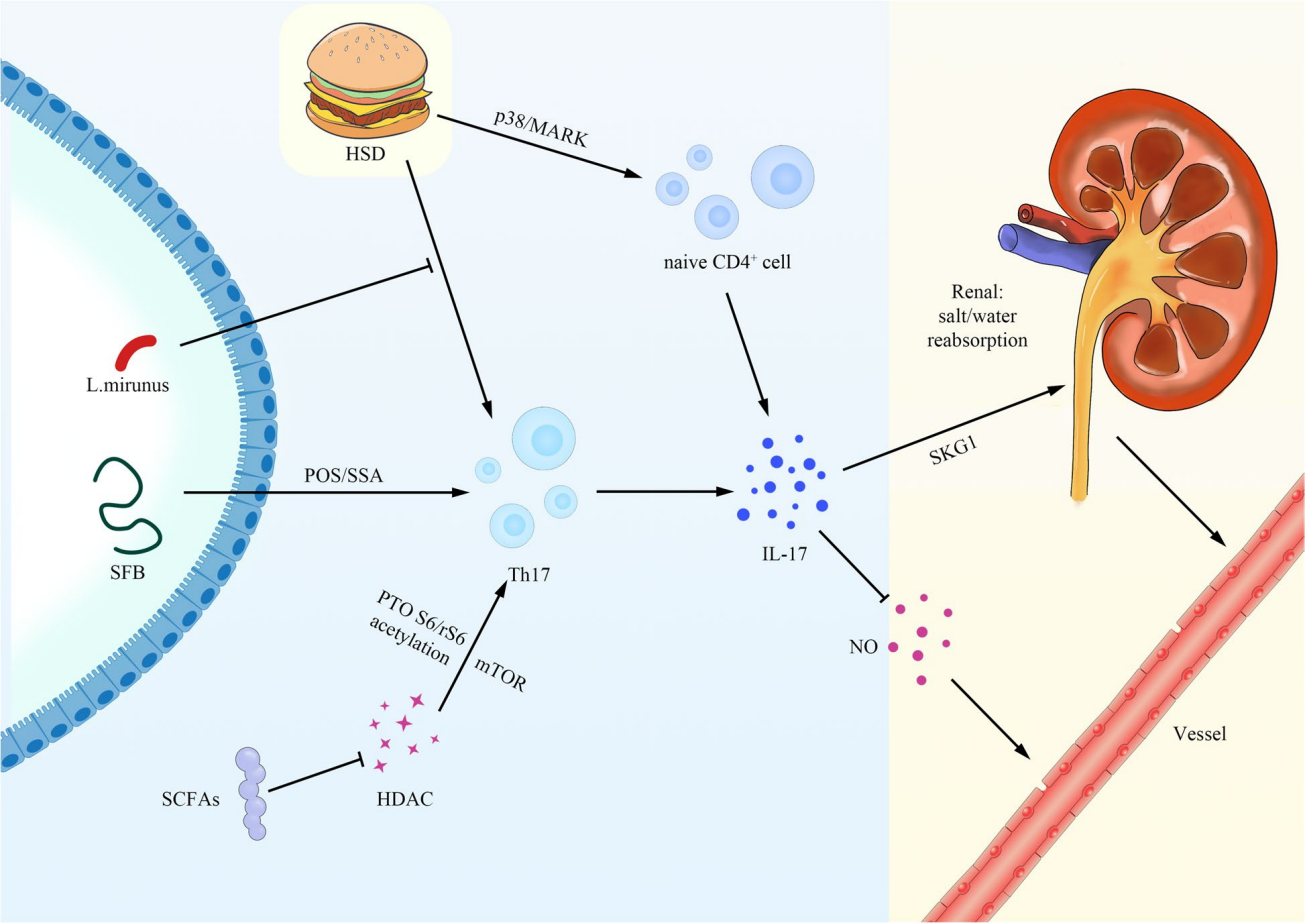
Model diagram of the regulatory of IL-17 between gut and hypertension. SCFAs can inhibit HDAC in T cells, which promotes the acetylation of kinase p70 S6 and phosphorylated rS6, thus promoting the mTOR pathway necessary for the proliferation of Th17 cells. HSD could activate the p38/MAPK pathway, involving TonEBP/NFAT5 SGK1 and subsequently mediate the secretion of IL-17a by naive CD4^+^ cells. Moreover, L.murinus can prevent production of Th17 cells induced by HSD. Th17 cells are also mediated by SFB through ROS and SAA. Then IL-17 can promote the reabsorbtion of salt/water and inhibit NO-derived from eNOS, consequently lead to elevation of blood pressure

Experiments of rats in vivo have also shown that the increasing salt (sodium chloride; NaCl) concentrations under physiological conditions could activate the p38/MAPK pathway, involving the activation of key regulators such as tension reactive enhancer binding protein (TonEBP/NFAT5) and SGK1 during Th17 polarization. This could specifically promote the Th17 cells generation and subsequently mediate the secretion of IL-17a by naive CD4^+^ cells in both mice and humans [88, 89]. Moreover, Th17 cells under high salinity showed a highly pathogenic and stable phenotype, characterized by up-regulation of pro-inflammatory cytokines GM-CSF, TNF-α and IL-2 [89]. Interestingly, Lactobacillus L.murinus can prevent HSD-induced production of Th17 cells and consequently ameliorate the salt-sensitive hypertension [90]. An experiment showed that lactulose supplementation in high salt diet (HSD)-fed mice increases the abundances of Bifidobacterium, Alloprevotella and Subdoligranulum by decreasing the IL-17a and IL-22 mRNA, leading to the relief of HSD-induced hypertension [91] (Fig. 2).

#### MCP-1 involved in vascular injury in hypertension

MCP, with a molecular weight of 8 ~ 10kD, is a protein family consisting of more than 10 kinds of proteins with large homology. The majority of the MCP genes have been mapped at human chromosome 17q11.2 [92]. MCP-1 is produced by many cell types, including endothelial, fibroblasts, epithelial, smooth muscle, mesangial, astrocytic, monocytic, and microglial cells [93]. MCP-1, also known as CCL2 and mainly utilizing CCR2 as its receptor, is a representative of the chemokine CC family (β subfamily) which can help chemotaxis monocytes and plays a key role in atherosclerosis, ischemia–reperfusion injury of the heart, heart failure and other cardiovascular diseases [94–96].

MCP-1 can promote the aggregation of monocytes and Mφ in the vascular wall and facilitate the release of inflammatory factors. This in turn leads to the hyperplasia of vascular smooth muscle as well as cell matrix deposition and consequently weaken the elasticity of the vascular wall in hypertensive patients [97, 98]. In wild type mice, AngII can increase circulating CCR2 intensity of mononuclear cells. However, this effect could be prevented by AngII type 1 receptor (AT1) blockers Olmesartan, as shown in AT1 receptor deficiency model mice [99]. At the same time, the CCR2 intensity on mononuclear cells is observed to be enhanced in the patients and rats with hypertension, suggesting that MCP-1 may regulate the functions of mononuclear cells through CCR2 and is related with vascular inflammation or AngII induced hypertension [100, 101].

In hypertensive mouse models induced by desoxycorticosterone acetate (DOCA)/salt, vascular accumulation of Mφ is reduced and blood pressure is lowered after CCR2 antagonist treatment such as INCB3344 [102]. Therefore, some researchers put forward that hypertension in mice may be associated with altered Mφ polarization and number or activation of the perivascular Mφ [103]. M2Mφ probably promotes extracellular matrix remodeling, so the use of CCR2 antagonist can reduce blood pressure through preventing monocyte/Mφ accumulation [104]. Besides, many studies showed the renal damage caused by Mφ influx or inflammation driven by MCP-1 [105, 106]. It can be seen that MCP-1/CCR2 signaling directs the vascular accumulation of monocytes/Mφ and leads to local injury. In addition, the infiltrating T cells and monocytes/Mφ could produce proinflammatory cytokines, such as TNF-α and IL-1β and further induce the expression of MCP-1 in the hypertensive tissue cells. Consequently, a positive feedback loop of MCP-1 and CCR2 is formed and the influx of immune cells conduces to the recruitment of more inflammatory cells, driving organ injury and dysfunction by hypertension [107].

Apart from the single effect of MCP-1 on hypertension, IL-17 may also participate in this process through promoting the secretion of MCP-1 in cardiac myocytes, fibroblasts, brain and other cells [108–111]. Further analysis showed that the activation of PI3K, JNK, ERK pathways may largely account for this process [112]. High glucose induces IL-17 expression via a PI3K → Akt → ERK-dependent signaling pathway and IL-17 could activate the Act1/TRAF6/TAK1 pathway to upregulate MCP-1 expression in the experimental autoimmune myocarditis [113, 114]. However, the detailed mechanism in hypertension remains unclear.

In addition, some studies showed that SCFAs can inhibit the expression of MCP-1 in human monocytes and peripheral blood mononuclear cells [115]. Researchers found decreased ROS in angiogenesis concomitant with the weakened mRNA level of MCP-1 after the infusion of AngII into GF mice. All these results indicate that the gut microbiota may promote vascular immune cell infiltration and inflammation driven by MCP-1, at least partly promote AngII induced vascular dysfunction and hypertension [116]. In addition, to suggest the role of gut microbiota in hypertension driven by MCP-1, more clinical or animal study is needed.

#### Effect of IL-6 on AngII-induced hypertension and differentiation of Th17 cells

IL-6 is a lymphoid factor mainly produced by activated T cells, bone marrow stromal cells, Mφ, dendritic cells, endothelial cells, fibroblasts, synovial cells, glia cells and keratinocytes [117]. It can cooperate with colony stimulating factors (CSF) to promote the proliferation and differentiation of primitive bone marrow cells so as to enhance the cleavage functions of natural killer cells. Surprisingly, some studies have also suggested its potential relationship with hypertension [118].

Experiments showed that the AngII induced hypertension was relieved in the IL-6^−/−^ mice [119, 120], suggesting a key role IL-6 plays in the induction of hypertension depending on JAK2/STAT3 pathway in the kidney [121]. Moreover, IL-6 is probably involved in hypertension considering its role in vascular inflammation, angiosclerosis and endothelial dysfunction [122]. In fact, apart from the direct promoting effect on hypertension, IL-6 is also reported to induce the differentiation of immature T cells into Th17 cells and is involved in the process of hypertension [123]. Whether CD4^+^ T cells differentiate into Th17 cells or Tregs depends on the local cytokine environment, and it has been shown that increasing IL-6 is necessary to drive Th17 cells differentiation and inhibit the development of CD4^+^ Tregs [124, 125].

At the same time, there is growing evidence that hypertensive stimulation increases the local IL-6 level in the cardiovascular system and kidney. AngII may contribute to norepinephrine release through central sympathetic nerve to mediate T cells activation and infiltration, stimulate the expression of IL-6 and result in the disproportionate differentiation of Th17/Tregs cells [126, 127].

Research found that intestinal microorganisms can promote the production of IL-6 by inducing DC and Mφ, and IL-6 get involved in regulating the effect of intestinal microorganisms on intestinal inflammation [19, 38, 39]. A study kefir, a probiotic obtained from the fermentation of milk by kefir grains reduced IL-6 and TNF-α protein densities in SHRs [128]. Using the human gut-on-a-chip microdevice to build the model of human intestinal inflammation and bacterial overgrowth, it was found that immune cells and lipopolysaccharide (LPS) endotoxin could jointly stimulate IL-6 secretion by epithelial cells [129]. Thus the intestinal environment or microbiota can affect the intestinal inflammation and influence hypertension through an IL-17-dependent approach. However, some researchers reckoned that it is microbiota-induced IL-1β, rather than IL-6, that is critical for the development of Th17 cells in the intestine [130]. Therefore, further researches are necessary regarding the specific role of IL-6 during Th17 cells differentiation.

#### Regulatory relationship of TGF-β with intestinal physiology and differentiation of Th17 cells

Transforming growth factor-β (TGF-β) belongs to a TGF superfamily including activins, inhibins, Mullerian inhibitor substance and bone morphogenetic proteins [131]. They regulate cell growth, differentiation and immune system and affect inflammation, tissue repair and embryonic development via different mechanism. In recent years, evidence has shown the relationship between TGF-β and the intestinal tract, as the production of TGF-β is regulated by intestinal microbiota [131].

In the gastrointestinal tract, intestinal epithelial cells (IEC) and immune cells are important sources of bioactive TGF-β. Epithelial cell injuries as well as the intestinal inflammation could enhance the production of TGF-β [132]. On the other hand, previous study demonstrated that blockade of TGF-β signaling does not alter proliferation of intestinal epithelial cells, but reduces intestinal epithelial cell migration [133, 134]. Present studies have demonstrated an increasing TGF-β production in mice colonized by clostridium difficile [135]. It is also worth mentioning that ATP produced by the microbiota can directly increase the production of TGF-β and indirectly increase the number of active TGF-β by promoting the expression of an integrin αvβ8 which is necessary for the activation of TGF-β on DC surface [132, 136]. Numerous studies in vitro also documented the ability of intestinal microbiota mediated TLR signaling in promoting the expression of αvβ8, confirming the promoting role of intestinal microbiota in TGF-β production and secretion [137]. All the evidence above suggests a close relationship between the intestinal microbiota and TGF-β.

Of significant interest, TGF-β also plays a key role in Th17 cells differentiation. In CD4^+^ T cells, TGF-β signaling promotes the expression of Rorc and Rorα in the presence of IL-6. They could respectively encode two transcription factors RorγT and Rorα, which are necessary for Th17 cells differentiation [138]. However, using flow cytometry to evaluate IL-17a^+^ cells and IL-17a secretion, early evidence suggests that the development of Th17 cells is significantly reduced in the absence of TGF-β [139]. Some studies further implicated that TGF-β coordinated the differentiation of Th17 and Tregs cells in a concentrations-dependent manner. At lower concentrations, TGF-β and IL-6 or IL-21 synergistically promoted Th17 cells differentiation, whereas at higher concentrations, TGF-β induced Foxp3 and thus facilitated Tregs lineage differentiation [140]. A new way to produce Th17 cells is also proposed in recent study, indicating that Th1 cells can be transformed into Th17 cells in response to TGF-β and IL-6, this trans-differentiation may occur in the intestinal tract and account for the higher level of Th17 cells in times of gastrointestinal inflammation [141].

At the same time, TGF-β, a key driver of renal fibrosis in hypertension, is associated with the RAS and could enhance the renal fibrosis by inhibiting the activation of matrix metalloproteinases (MMPs) that increases extracellular matrix deposition [142]. Taken as a whole, these results suggest that TGF-β is a noteworthy factor in organ damage during the hypertension.

#### IL-10 as a bridge between hypertension and intestinal microbiota

IL-10, MW as 20.5 kDa, is a vital immune regulatory cytokine that acting on various types of immune cells. It has profound anti-inflammatory functions and could limit the excessive tissue disruption caused by inflammation. Studies have confirmed the production by various types of immune cells In humans, including CD4^+^ and CD8^+^ T cells, B cells, Mφ, DC, neutrophils, natural killer cells and eosinophils [143].

Former studies have proposed that IL-10 production in myeloid cells was triggered by microbial products through pattern recognition receptors (PRR) [144]. The intestinal microbiota increased the number of T cells and B cells and activated IL-10 production by intestinal B cells in a MyD88/TLR2/PI3K dependent manner [145, 146]. In the meantime, researchers documented the production of IL-10 by resident intestinal Mφ, whose homeostasis is also regulated by the direct stimulation of dietary amino acids [147]. SCFAs also promote the antigen-specific Th1 cells expression of Prdm1 which encode B lymphocyte-induced maturation protein 1 (Blimp-1), a protein necessary for T cells to produce IL-10. This is mediated by G-protein coupled receptor 43 (GPR43) and could regulate the consequently reducing level of IL-10 in tissue injuries [148–150]. Yet in another experience, SCFAs can specifically inhibit the production of LPS-induced IL-10 in human monocytes without affecting the secretion of other cytokines or chemokines examined [115]. Taken together, a regulatory relationship between intestinal microbiota and IL-10 could be established which show beneficial influences on hypertension, suggesting that diversity of intestinal flora may ameliorate healthy state.

All cytokines within the IL-10 family signal through receptors belonging to the type II cytokine receptor family. IL-10 acts as a major anti-inflammatory factor, leading primarily to the activation of the IL-10/JAK1/STAT3 signal transduction cascade reaction [151]. In hypertension, to further elucidate the role of cardiovascular protective effect of IL-10, several studies indicate that the carotid artery of IL-10^−/−^ mice exposed to AngII experienced conspicuous endothelial dysfunction with a higher level of blood vessels superoxide, while artery of wild type mice was relatively less influenced [152]. After injection with Ang II, in vitro studies revealed a higher level of the mean arterial pressure (MAP) in IL-10^−/−^ mice, accompanied with vascular dysfunction and the formation of superoxide [153]. As anticipated, the hypertensive rats treated with IL-10 exhibited an improvement of endothelium-dependent diastolic functions by inhibiting NADPH oxidase activity in Ang II [154].

**Fig. 3:**
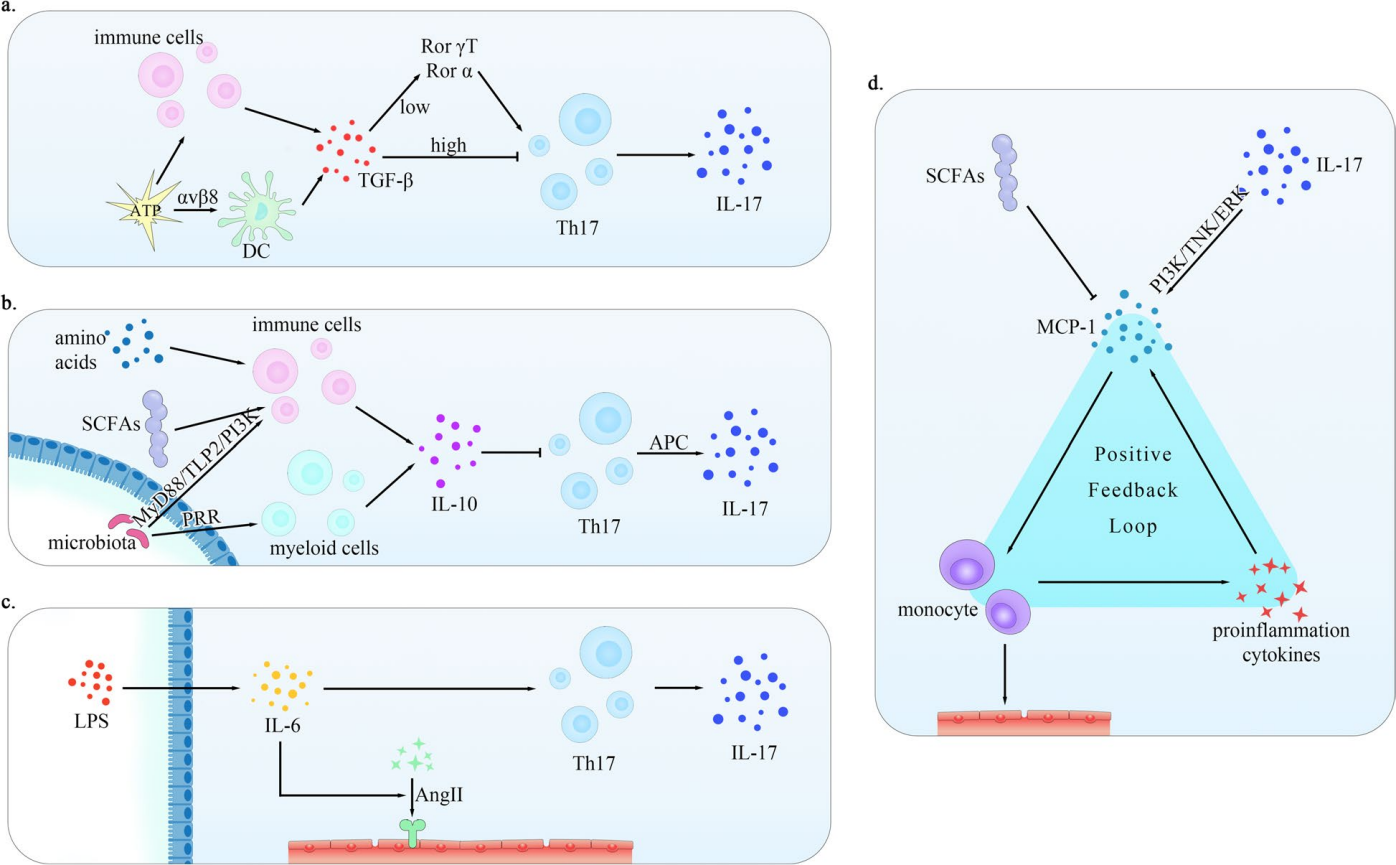
Correlation pattern of MCP-1, IL-6, TGF-β and IL-10 between gut and hypertension. **a** ATP can promote the secretion of TGF-β by immune cells and DC in different ways, and TGF-β could further drive the differentiation of Th17 cells. However, TGF-β has an inverse effect at high concentrations **b** Th17 cells can be inhibited by IL-10 in an APC-depended way. IL-10 is secreted by several cells like immune or myeloid and mediated by gut microbiota and metabolism. **c** IL-6 can be produced by LPS-stimulated IEC and involved in hypertension induced by AngII. **d** SCFAs can inhibit the expression of MCP-1, which promotes the aggregation of monocytes as well as Mφ and lead to a lower elasticity of vascular wall. Monocytes and Mφ also produce proinflammatory cytokines that induce the secretion of MCP-1 and thus form a positive feedback loop. Additionally, IL-17 induces MCP-1 production in PI3K, JNK, ERK pathways

As shown in Fig. 3, although the biological function has not been defined, IL-17 also seems to play a role in the process of IL-10 regulated hypertension, because IL-10 could suppressed the generation of Th17 cells while enhance the number of regulatory T cells [155]. Latest researches reveal that IL-10 secreted by antigen presenting cells (APC) is a determinant in preventing the onset of TH1/Th17-mediated inflammation in bacterial antigen-driven CD4^+^ cells [156]. The production of IL-17 was inhibited by IL-10 [157, 158]. However, this inhibition is not through the direct action on Th17 cells because of diminished cytokine receptivity, but rather depends on the APC [159, 160]. Taken as a whole, these results confirmed the protective role of IL-10 and its relieving effect on blood vessels and hypertension.

##### Box 3 important immune factors in hypertension

IL-17 can promote hypertension through several immune factors, and become the main connection between intestine and hypertension.

The expression or secretion of MCP-1, IL-6, TGF-β and IL-10 are influenced by microbiota or its metabolite and have potential effect on hypertension.

The immune factors mentioned form an IL-17-centered regulatory relationship through impacting Th17 cells.

## Conclusions and future perspectives

Based on the analysis above, these findings have significant implications for the understanding that the immune system represents a remarkable role in hypertension. Intestinal microbiota and its metabolites form a complex immune regulatory relationship through various cytokines in the intestinal tract and participate cooperatively in the process of human hypertension. Recent evidence supports that gut microbiota can protect or promote the development of hypertension by interacting with gut secondary lymph organs and altering Th17 cells and IL-17, with subsequent changes in T cells infiltration in vascular tissues. IL-17 is considered to be the most important approaching molecule in this process. Therefore, the inhibition of IL-17 has a prospect for relieving hypertension and deserves further investigations. Other factors such as MCP-1, TNF-β, IL-6, and IL-10 also show potential for blood pressure regulation and maybe the accessories to IL-17 (summary see Box 2). In addition to controlling the rise of blood pressure, the treatment of hypertension also requires the protection of organs and tissues affected by it.

Meanwhile, the role of intestinal microbiota in hypertension requires further investigation. Considering the enormous amount of intestinal microbiota hosted in the body, the detailed processes and type specificity of microbiota in the process of hypertension remain poorly understood. What’s more, MCP-1, IL-6, TNF-β and IL-10 deserve the attention of cardiovascular experts due to their effects on kidney, cardiovascular and other tissues. Therefore, the intestinal tract might be more a novel approach for the treatment of hypertension. In addition, IL-17 is also involved in several other diseases (see Table 1), indicating the potential ability and value of IL-17, and deserves further studying.

**Table 1:** To summarize the mechanism of IL-17 in different diseases

Disease	Mechanism	Refs
Aortic dissection	Reduced endothelial progenitor cells partly related to upregulated IL-6/IL-17 through DiI-acLDL/lectin and CD34^+^KDR^+^ cells	[161]
Cardiovascular diseases with (dyslipidemia, hypertension and diabetes mellitus)	Enhancement of IL-17 /IFN-γ production of CD4^+^28^null^NKG2D^+^ T cells	[162]
Type 2 Diabetes Mellitus	IL-10/IL-17 correlated negatively with BMI or metabolic index	[163]
Preeclampsia	Mediated placental ischaemia and oxidative stress though AT1-AA production	[164, 165]
Idiopathic pulmonary arterial hypertension	TH17 cell immune polarization and IL-17A with disease severity or outcome associated conditions	[166, 167]
Systemic sclerosis	Activation of Th17 and Th1 cell responses	[168]
Schistosomiasis japonica	Activation of γδT cells and producing IL-17 against parasite eggs	[169]

Moreover, immune factors as the potential treatment are mainly limited to extensively side effects, while the gut microbiota and its metabolites as potential markers and mediators of hypertension are relatively infertile. This may be due to both the complexity and multifactorial etiology of hypertension and the lack of molecularly targeted investigations on drug development. However, more recently, with the advent and evolution of metabolomics’ technologies, more discoveries of active metabolites would be of great help in understanding the underlying mechanisms in disease pathologies.

## Data Availability

Not applicable.
